# Lived experience of participants who engaged in the co‐creation of initiatives to improve children's health in a rural Australian community

**DOI:** 10.1111/ajr.12996

**Published:** 2023-05-18

**Authors:** Carmen Vargas, Monique Hillenaar, Claudia Strugnell, Steven Allender, Lucy Marks, Melanie Green, Carolina Venegas Hargous, Michelle Jackson, Colin Bell, Jillian Whelan

**Affiliations:** ^1^ School of Health and Social Development, Institute for Health Transformation Deakin University Geelong Victoria Australia; ^2^ Mansfield District Hospital Mansfield Victoria Australia; ^3^ School of Medicine, Institute for Health Transformation Deakin University Geelong Victoria Australia

**Keywords:** co‐creation, community‐led, healthy eating, obesity prevention, physical activity, rural health

## Abstract

**Objective:**

To describe participants' lived experience of co‐creating and implementing initiatives to improve children's health.

**Design:**

This manuscript reports an embedded case study design, which aims to describe participants' lived experiences of co‐creating community‐based initiatives. Information was gathered from an online survey and two focus groups. The two transcribed discussions from the focus groups were analysed using a 6‐step phenomenological process.

**Setting:**

Mansfield, Australia, population 4787, is one of ten local government areas (LGA) participating in the Reflexive Evidence and Systems Interventions to Prevent Obesity and Non‐communicable Disease (RESPOND) project.

**Participants:**

Participants were purposively selected from established community groups previously engaged by RESPOND using a co‐creation approach. The recruitment for the focus groups was a convenient sampling from participants that provided their email addresses in the online survey.

**Results:**

Eleven participants completed the online survey. A total of ten participants attended the two focus groups of 1‐h duration: five participants in each. Participants reported feeling empowered to create unique, locally relevant and readily adaptable community‐wide change. They were supported by a strong partnership that mobilised funding for a part‐time health promotion employee. Strengthened social connections were an unexpected though highly valued outcome.

**Conclusion:**

Co‐creation processes may assist stakeholders in delivering prevention strategies in ways that are empowering for them, responsive to the changing needs of the community, strengthen organisational partnerships and enhance community participation, social inclusion and engagement.

**What is already known on this subject**

- Multiple studies have called for a systems approach to various chronic disease prevention efforts, but few have identified how to operationalise it.
- Multiple studies have identified that true engagement with community members enhances the potential for positive and sustained change.
- Effective co‐designed or co‐created approaches to prevention and health promotion have the potential to empower communities to lead and sustain change.
- Co‐design positions the expertise and knowledge of individuals at the centre of the creation of aims, plans and outcomes.

**What this study adds**

- Insights from the perspective of participants engaged in co‐creation, experiences of the process and impacts of their role in promoting health within the community.
- Contributes to the co‐creation literature in public health by providing a specific case study that shows the way principles of co‐creation are experienced in public health systems initiatives.

## 1 INTRODUCTION

Modern societies face multiple complex systemic problems such as climate change, chronic diseases, health and social inequity and homelessness.^1^ Children above a healthy weight are at an enhanced risk of multiple chronic diseases in childhood, decreased quality of life^2^ and ongoing health issues, as unhealthy weight often tracks into adulthood.^3^ Recent public health guidance has pointed to the use of systems science to underpin the next generation of interventions.^4^ At the core of any resolution to such complex issues is the effective engagement of multiple stakeholders who bring different knowledge, skills, insights and experiences to understand the key drivers of the problem and to develop potential solutions specific to the context.^5^

One trial attempting to co‐create community‐led systems change the Reflexive Evidence and Systems interventions to Prevent Obesity and Non‐communicable Disease (RESPOND) project.^6^ RESPOND is a stepped wedge cluster‐randomised control trial that aims to promote children's health across 10 rural and regional LGAs in northeast Victoria, Australia. It involves a community‐led whole‐of‐the‐systems approach to childhood obesity prevention^6^ that utilises a series of group model building (GMB) workshops^7^ to develop a shared understanding of the local causes of unhealthy weight in local children. Once the key drivers of the complex problem are identified, stakeholders prioritise and co‐design locally relevant actions that community stakeholders implement.^8^ Traditionally, these ‘actions’ comprise a variety of initiatives that act at different parts of the system and aim to change local children's physical activity, food environments and health behaviours.^9^ Typically, participants are engaged in small ‘working groups’ or ‘implementation teams’ beyond the GMB process to implement the prioritised actions.^10^

It has been argued that public health interventions should be considered ‘actions’ within complex systems. This view helps to describe ways to work in collaboration within the system to create change.^11^ Co‐creation, which involves engaging multiple stakeholders across all project phases, has been shown to increase intervention effectiveness and, combined with systems approaches, envisages enhancing intervention design, implementation and evaluation.^12^ In this way, co‐creation can serve as an avenue to provide a systematic approach to active collaboration.^12^ Yet few insights are available on the drivers and dynamics of co‐creation, how participants experience and value this participation, as well as outcomes for communities.^13^ Within co‐creation, interactions between stakeholders (e.g. volunteers, community leaders, health promotion officers, etc) can lead to the creation of value and optimisation of resources (i.e. financial, skills, knowledge, time).^14^ The techniques to facilitate this engagement have implications for the lived experience of participants involved in this process and the effectiveness of any co‐designed outputs.^15^

Conceptually, the broader term ‘co‐creation’ encompasses principles of effective engagement from the identification stage of a problem to the co‐design of potential solutions, implementation, evaluation and continuous improvement cycles.^16^, ^17^, ^18^, ^19^ Co‐design under the umbrella of co‐creation aims to promote citizen engagement and participation in the process and outcomes of a project.^20^, ^21^ For effective co‐design, a broad base of expertise and knowledge is essential in the design process.^20^ Beyond collaboration, co‐design emphasises equal and reciprocal relationships between all involved parties.^22^ It is theorised across several co‐created initiatives that new relationships emerge and existing relationships are strengthened to co‐create stronger initiatives that can change social norms and environments.^8^, ^23^, ^24^ Despite this potential of co‐creation, few studies have reported how these stakeholders experience co‐creation, particularly within a large, funded trial. This study is an in‐depth exploration of the lived experience of co‐creation within one of ten LGAs within the RESPOND project.^25^

This manuscript sets out to answer the following research question:

1. What was the lived experience of participants engaged in a co‐creation process during the Mansfield‐RESPOND project?

## 2 METHODS

### 2.1 Study design

**FIGURE 1:**
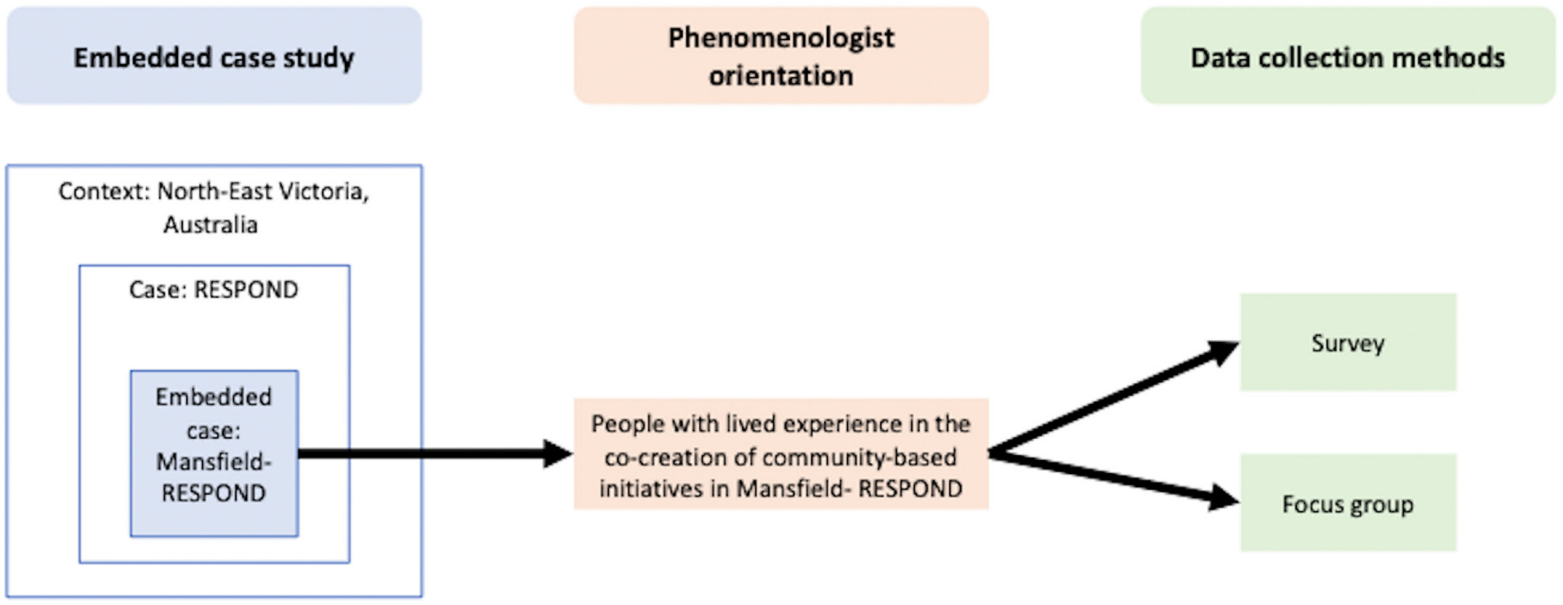
Study design, epistemology and methods.

This study can best be described as an embedded case study design^26^ based on a phenomenologist orientation^27^ (Figure 1). Phenomenology calls for the participation of people with lived experience of a phenomenon of interest. A phenomenological orientation was used to elucidate the socially constructed meaning of and lived experience of the co‐creation process.^27^ This case study is about people who have lived experience in co‐creating community‐based initiatives and involves one community within the RESPOND project (Mansfield RESPOND) as the embedded unit of analysis. To document the case study, data were collected using an online survey and focus groups; this combination of methods enhanced the understanding of the case study. The study design and reporting of results and analysis were informed by the guidelines for conducting and reporting mixed research in counselling and beyond.^28^

### 2.2 Setting: Mansfield ‐RESPOND background

Mansfield, Australia, is one of 10 rural and regional Victorian LGAs within the RESPOND project. Mansfield is a small rural town situated near the base of Mount Buller, approximately 190 km northeast of Melbourne. Mansfield has a population of 4787 comprising 927 children aged 0–14 years (0–4 years: 286, 5–9 years: 362, 10–14 years: 279).^25^ Mansfield has a lower median weekly household income of $1088 compared to the Victorian median weekly income of $1419.^25^

Data collected in 2019 within Mansfield showed 21%–25% of primary school children were above a healthy weight, 90% did not meet daily fruit and vegetable recommendations, and active transport was lower than in metropolitan areas. This data mobilised the community to engage with RESPOND. Local facilitators trained in the use of community‐based systems thinking helped community members through two group model building (GMB) workshops, to co‐design a causal loop diagram (CLD) representing the systemic drivers of: ‘*What helps and what discourages our children to eat healthily and be physically active*?’. Participants then co‐created and implemented initiatives designed to improve children's health. This study will report on the lived experiences of those involved in co‐creating community‐based initiatives in Mansfield.

### 2.3 Participants recruitment

Researchers (MA and MG) used purposive sampling to identify key local stakeholders from the Mansfield Health service, Mansfield Shire Council, community members, including parents/carers of young children, school representatives, the fitness industry and volunteers. These stakeholders were invited via email to complete an online survey. The recruitment for the focus groups utilised convenience sampling from participants that provided their email addresses in the online survey. These were invited via email to be involved in a 1 h online (Zoom) focus group.

### 2.4 Data collection

#### 2.4.1 Online survey

An online survey comprised 24 questions. The survey was designed to examine the lived experience of co‐creation through RESPOND. It was drawn from published studies on co‐creation, co‐design and co‐production.^14^, ^29^, ^30^ The survey (available as Appendix S1) captured general information on study participants (i.e. gender, age group, and role in the community), their motivation for joining the project (measured through an open‐ended question and a scale from 1 to 10, 1 being low and 10 being high), the phases of the project in which participants were involved, and their lived experience of collaboration and communication in this process. This was measured through a five‐point Likert scale, where participants specified their level of agreement with 14 statements regarding co‐creation aspects of the process (Strongly Agree, Somewhat Agree, Neither Agree nor Disagree, Somewhat Disagree and Strongly Disagree).

#### 2.4.2 Focus group

The focus groups captured information on participants' lived experiences of the various phases of co‐creation in RESPOND. Principles of co‐creation, co‐design and co‐production guided the development of the interview schedule^14^ (available as Appendix S2). The focus groups were recorded with participant consent, transcribed verbatim and emailed to participants for approval. Comments were received from three participants, and the transcripts were subsequently adjusted.

### 2.5 Data analysis

Quantitative survey data were analysed using simple descriptive statistics (e.g. frequency and proportions to response options). Responses to questions answered on a scale from 1 to 10 were categorised as low (1–3), moderate (4–7) and high (8–10). Likert scale questions were analysed in conjunction with the focus group material. For open‐ended response options, thematic analysis was used to group responses into categories and categories were quantified. Simple descriptive statistics were used to report these results. NVivo12 (Windows)^31^ was used to organise the focus group data. Researchers (MH and JW) followed the seven‐step data analysis method developed by Colaizzi for phenomenological research,^32^ which involved: (1) reading and rereading the transcript; (2) extracting significant statements that pertain to the phenomenon; (3) aggregating formulated meanings into theme clusters and themes; (4) formulating meanings from significant statements; (5) developing an exhaustive description of the phenomenon's essential structure or essence; (6) a description of the fundamental structure of the phenomenon is subsequently generated; (7) validation of the findings of the study through participant feedback completes the analysis.^32^

## 3 RESULTS

### 3.1 Participants

The survey was emailed to 25 stakeholders in January 2022, of which 11 (44% participation rate) participants completed the online survey. A total of 10 out of the 11 survey participants indicated their willingness to participate in the focus group, with 10 attending (91%). Two 60‐min focus groups, with five participants each, were conducted by JW and MH online via Zoom. All participants were female and represented a wide range of age groups, with five (46%) participants in the 25–44 age gap, four (36%) in the 45–64 age group, and two (18%) in the 65 years or over. Participants represented all the organisations and groups originally invited (Mansfield Health Service, Mansfield Shire Council, community members including parents/carers of young children, school representatives, fitness industry and volunteers).

Participants were asked to describe their motivation (reasons) to join the Mansfield RESPOND, 72% (*n* = 8) of the answers represented an extrinsic reason with a strong interest in helping and supporting the community. The level of motivation for joining the Mansfield RESPOND project was examined on a 10‐point Likert scale, with 1 being low and 10 being high. Most participants (82%, *n* = 9) reported their level of motivation as high (8–10). In the same way, participants were asked about their current level of motivation for being involved in the Mansfield RESPOND project. Most participants were involved in at least one GMB workshop (72%, *n* = 8) as part of Mansfield RESPOND. Regarding the phases of the project in which they were involved, all participants had an important role throughout the project. Table 1 provides a full description of the survey answers.

**TABLE 1:** Summary of participants motivations and involvement.

	% (*n*)
1. Motivations to join the Mansfield RESPOND Project (participants were allowed to do multiple entries)	
Achieve better health outcomes to the community	46 (5)
Achieve good nutrition and access to healthy foods	36 (4)
Promote physical activity within the community	9 (1)
Involved as part of their employment	27 (3)
2. Level of motivation for joining the Mansfield RESPOND	
Low	9 (1)
Moderate	9 (1)
High	81 (9)
3. Current level of motivation on the Mansfield RESPOND	
Low	–
Moderate	9 (1)
High	81 (9)
N/A	9 (1)
4. Participation in GMB workshops as part of Mansfield RESPOND	
None	27 (3)
GMB 1	36 (4)
GMB 2	64 (7)
GMB 3	45 (5)
5. Phases of the project in which participants were involved (participants choose as many as relevant)	
Identifying the problem	64 (7)
Analysing the major drivers	55 (6)
Defining the actions to address the problem	45 (5)
Designing and planning actions	55 (6)
Implementing	73 (8)
Evaluating	45 (5)

Overall, participants described the co‐creation process as a positive experience, with ≥90% of respondents indicating that Mansfield RESPOND was useful for community stakeholders. When analysing the two focus groups together, four themes were identified (1) empowerment; (2) co‐creation of solutions; (3) partnerships and resources; and (4) social outcomes. These themes are discussed below alongside relevant survey data, as summarised in Table 2.

**TABLE 2:** Summary of Survey Results aligned with Focus Group Themes: Lived Experience of Mansfield RESPOND.

To what extent do you agree or disagree with the following statements about the Mansfield RESPOND project?	*N*	Strongly disagree % (*n*)	Somewhat disagree % (*n*)	Neither % (*n*)	Somewhat agree % (*n*)	Strongly agree % (*n*)
**1. Empowerment**						
It respected and valued the knowledge of each participant	11	—	—	—	45 (5)	55 (6)
2 It maintained respect for all the participants	11	—	—	—	45 (5)	55 (6)
3 It allowed participants to share their power to make changes	11	—	—	—	55 (6)	45 (5)
4 It helped stakeholders share their experience	11	—	9 (1)	9 (1)	36 (4)	45 (5)
**2. Co‐creation to address community needs**						
It allowed participants to design actions together	11	—	—	—	27 (3)	73 (8)
2 It allowed the interchange of ideas during the implementation process	11	—	—	—	45 (5)	55 (6)
3 It helped to understand interactions between participants	11	—	9 (1)	9 (1)	45 (5)	36 (4)
4 It included all participant's perspectives and skills	11	—	9 (1)	—	64 (7)	27 (3)
5 It allowed equal collaboration between the people involved	11	—	—	9 (1)	64 (7)	27 (3)
**3. Partnerships and resources**						
It created a way to keep an ongoing conversation between stakeholders	11	—	—	18 (2)	18 (2)	64 (7)
2 It allowed an open communication	11	—	—	—	4 (7)	(4)
**4. Social outcomes**						
It contributed to building and maintaining relationships between participants	11	—	—	9 (1)	45 (5)	(5)
**5. Other**						
It was useful for community stakeholders	10	—	10 (1)	—	30 (3)	60 (6)
2 It allowed an empathetic communication	11	—	—	9 (1)	45 (5)	45 (5)

#### 3.1.1 Empowerment

Regardless of whether they represented an organisation or were volunteers, participants described the process of co‐creation as empowering. They described it as giving a voice to the community, providing an opportunity to feel heard, and they felt supported to bring their solutions to life. Organisation representatives identified a strong sense of work satisfaction that arose because of this co‐creation work with the community.

when you bring the community with you, it's a much more rewarding experience (F1, P3)

Several reflected on the satisfaction of working with their community:

We actually listened to the community, and we achieved what the community asked. And that to me was a magnificent achievement … and to see what we've achieved in this short space of time is just extremely rewarding. (F2, P2)

Similarly, community stakeholders spoke of the value they felt in being listened to and heard:

what I really got out of this and helped [it] get going; was allowing the voice of the volunteers, and for that volunteer to be empowered… when you're on this project having a brainstorming session, it gives you empowerment and excitement in being heard (F1, P2)

All survey respondents (100%) either somewhat or strongly agreeing that the Mansfield RESPOND project respected and valued the knowledge of each participant, maintained respect for all participants and allowed participants to share their power to make changes, and 81% either somewhat or strongly agreeing that it helped stakeholders share their experience.

#### 3.1.2 Co‐creating community needs

Most participants emphasised that co‐creation led to chosen initiatives that reflected what the community most wanted and needed and identified the value of having a community‐led approach as opposed to a top‐down approach:

being the community‐led projects, I feel there's so many more ideas that come through… rather than being dictated what has to be done. (F1, P4)

and

that's the difference from, I suppose, coming up with an idea in an office than coming up with an idea of the community group of people that are on the ground, living the experiences and living in the community (F2, P3)

The initial action ideas co‐created within the GMB workshops were adapted where necessary by community stakeholders as they learned what was working and what could be tweaked. They changed strategies to improve the fit of the initiative to the community. These adaptations were in part due to the timing of the initiative during the COVID‐19 pandemic. Stakeholders reflected that they were able to adapt to COVID‐19 disruptions because of the established and existing passion of participants.

We've been able to adapt with the community really quickly to make changes, to enable things to work better. (F1, P3)

Participants acknowledged initiatives did not always go well; in these cases, it was within their power to adapt:

Some things didn't work, and that's okay. But we were like, ‘Okay, well, if we're going to do that again next year, how could we do it differently?’ (F2, P3)

Participants reported that often their health promotion work involved ‘reacting’ urgently to an issue. The co‐creation process, on the other hand, allowed them to be proactive:

I think it was nice to be a part of an initiative that was proactive rather than reactive. (F2, P4)

All survey participants (100%) either somewhat or strongly agreed that the Mansfield RESPOND project allowed participants to design actions together and allowed the interchange of ideas during the implementation process. Most participants either somewhat or strongly agreed that the process helped them understand interactions (81%), included all participants' perspectives and skills (91%) and allowed equal collaboration between people (91%).

#### 3.1.3 Partnerships and resources

Participants reported that co‐creation resulted in a strengthened partnership between the two lead agencies, the health service and local government. They considered the support of this partnership essential for implementing the initiatives: *‘to get there, you actually have to have the organisation behind you, backing you’ (F2, P1)* and *‘I would say yes, it's probably the most united I've seen it’ (F2, P3)*.

In exploring the history of this partnership, stakeholders reported that community consultation had occurred on previous occasions but had not been actioned. This experience motivated a key stakeholder into the direction required needed to move forward:

if we go back out and pull a group of the community together, then we needed to really commit to seeing some outcomes and working with them. (F1, P3)

Similarly, community volunteers reflected on their own shared commitment and partnerships: *‘we got a lot of buy‐ins’ (F2, P2)* and *‘it all sort of clicked’ (F1, P5)*. They also identified the value in the shared decision‐making that enabled effective action, not simply empty promises: *‘So bringing all of that together, I think it really, and as [participant 4] said, there's probably been a lot of, we're going to do this, and we're going to do that, but we've actually action[ed] it and come through with the goods.’ (F2, P3)*.

There was consensus within both focus groups that the resource of a dedicated health promotion worker (.3FTE) was essential as *‘the driving force and just kept the motivation going’ (F1, P1)* and ‘*it would not have worked if we didn't have person x and person y’ [referring to the health promotion worker and their manager] (F1, P2)*. A key organisation understood the importance of embedding resources into existing roles: ‘*[One partner] actually went back and re‐named [partner colleague]'s position to include health promotion so that it was valued’ (F2, P1)*.

Survey results showed that 82% of participants either somewhat or strongly agreed that the Mansfield RESPOND project created a way for ongoing communication between stakeholders, and 100% of participants either somewhat or strongly agreed that it allowed open communication.

#### 3.1.4 Social outcomes

Unplanned co‐benefits arose from the co‐creation methodology that strongly aligns with principles of improved social connection and strengthened social cohesion.

These social outcomes were observed generally:

I thought it was a great initiative to get people out and about, and the people that attended sessions were not people that you would see in the community regularly, so you were meeting new people as well, which was great. (F1, P4)

As well as, more specifically, reflecting on individuals who perhaps held loose ties to the community:

that was a way of socially connecting and giving, and it was one of the older men in our community, and I just thought that it was just absolutely wonderful that he probably wouldn't have had those discussions and chats with the group of volunteers without having something to give, so it gave a real purpose (F1, P3)

Participants reflected on how the process had extended their social connections, interactions, and conversations. For example: *‘now we've got normal conversations coming through from all walks of life’ (F2, P4)* and *“… none of my friends are really involved in the program, so I've met a lot of new people… Sorry, ‘none of my friends’, they have become friends as a result of it’” (F1, P2)*.

Survey results showed that 91% of participants either somewhat or strongly agreed that Mansfield RESPOND contributed to building and maintaining relationships between participants.

## 4 DISCUSSION

This study provides insights into participants' experiences of the co‐creation of a children's health initiative. Participants described the co‐creation process as beneficial for themselves and the community. They identified these benefits as empowerment, responsiveness to changing community needs and wants, a strengthened and productive partnership between two key local organisations (health service and local government), and wider and more meaningful community engagement than they had experienced previously. These findings contribute to the limited research that has examined the lived experience of co‐creating an initiative from a participant perspective, particularly within a large‐scale funded trial.

The dynamic exchanges between participants in our study activated and empowered them to improve the whole community's health. This finding of empowerment was also identified as an outcome of co‐creation in a recent scoping review that linked complexity‐informed health promotion with co‐creation across 27 health promotion initiatives.^33^ Linked with empowerment are concepts of motivation for engagement with the change process.

Our findings of a generally high and sustained level of motivation reported by our participants align with concepts reported by other studies.^34^ Community‐centred motivation is enhanced where participation in co‐creation promotes social change. Maintaining this social exchange is essential to keep participants engaged in the co‐creation effort.^34^ This could explain the high level of engagement and “passion” reported by participants in our study who observed a process of social change and social outcomes. Aligning participant motivations may increase the creation of value and enhance longer‐term outcomes.

The importance of effective partnerships has previously been documented in community change projects^35^ and was also observed by participants in this study. Any partnership may be at risk of issues of power imbalance; partnerships engaged with co‐creation efforts are no exception.^36^ Our results show that the partnership between the key agencies and the united front to the community minimised these potential problems of power imbalance. As emphasised by Smith (2018), an equal partnership between designers and end users is important for successful co‐creation.^37^ The strong partnership also ensured health promotion professionals were identified as passionate, competent and supportive community stakeholders. The process Mansfield RESPOND engaged within the identification and alignment of motivations enabled the optimisations of stakeholders' priorities and, thereby, the design of initiatives that co‐created value^34^ for the participants and the broader community.

For co‐creation, interactions between stakeholders can lead to value creation and optimisation of resources (i.e. financial, skills, knowledge, time),^14^ as in our study where resources were mobilised from key agencies, volunteer resources were engaged, and strong community connections were forged.

### 4.1 Strengths

This paper is about the lived experience of co‐creation, and two of the authors of this paper, work within this initiative. We have gathered insights directly from a breadth of health practitioners and community stakeholders involved in a co‐creation process with community members, local organisations and researchers. This case study provided a depth and breadth of understanding of the lived experience of co‐creation using a mixed methods approach (quantitative and qualitative).

### 4.2 Limitations

The study reports a low response rate from the survey and is from one of the 10 communities in RESPOND. Representation was not seen from the service club and sporting club disciplines. However, every other discipline had at least one representative participate, suggesting that a variety of perspectives was gained, and it achieved the goal of qualitative research providing depth rather than breadth. From a phenomenological approach, the analysis here is impacted by the authors' experience. These experiences are described in the reflexivity section above. This study was conducted retrospectively and therefore was dependent on participants' memory of events; however, this is a problem common to all research. This study is current, and participants are still engaged with the work. Future studies should try to engage larger participant groups.

### 4.3 Implications for practice

It is possible to empower communities and engage them in the co‐creation of initiatives to promote healthy eating and active living in children. Meaningful engagement is appreciated and leads to actions that better meet the needs of the community and provide what the community identifies as their needs and wants. For true and successful co‐creation to occur, a commitment is required from locally funded resources to facilitate the process, at least in the initial phase of a project, until community champions are upskilled and empowered to lead change on their own.

### 4.4 Future research

Further research is needed to replicate these findings to determine if this is a common experience or unique to this community. It would be useful to explore the links between levels of community empowerment and trial outcomes and identify if these short‐term findings translate into the long‐term impacts of co‐creation on rural communities.

## 5 CONCLUSION

Co‐creation processes may assist community organisations and volunteers in delivering prevention strategies in ways that are empowering for them, responsive to the changing needs of the community, strengthen organisational partnerships and enhance community participation, social inclusion and engagement.

## AUTHOR CONTRIBUTIONS

**Carmen Vargas:** Conceptualization; investigation; methodology; validation; visualization; writing – original draft; writing – review and editing; formal analysis; project administration. **Monique Hillenaar:** Investigation; writing – original draft; methodology; writing – review and editing; formal analysis. **Claudia Strugnell:** Supervision; investigation; funding acquisition; writing – review and editing. **Steven Allender:** Funding acquisition; writing – original draft; writing – review and editing; methodology; supervision. **Lucy Marks:** Investigation; conceptualization; writing – original draft; writing – review and editing; project administration. **Melanie Green:** Conceptualization; investigation; writing – original draft; writing – review and editing; project administration; supervision. **Carolina Venegas Hargous:** Writing – original draft; methodology; writing – review and editing. **Michelle Jackson:** Writing – original draft; writing – review and editing. **Colin Bell:** Conceptualization; funding acquisition; writing – original draft; writing – review and editing; methodology; formal analysis; project administration; supervision. **Jillian Whelan:** Conceptualization; investigation; writing – original draft; writing – review and editing; validation; methodology; formal analysis; project administration; supervision.

## CONFLICT OF INTEREST STATEMENT

This research has not been submitted elsewhere for publication. There are no conflicts of interest to declare. JW is supported by a Deakin University Deans Postdoctoral Research Fellowship. JW and CV are supported by, and SA and CB are named investigators on the National Health and Medical Research Council (NHMRC) funded Centre of Research Excellence in Food Retail Environments for Health (RE‐FRESH) (APP1152968). The opinions, analysis and conclusions in this paper are those of the authors and should not be attributed to the NHMRC. MH and MJ are supported by, and SA, CS and CB are investigators on the National Health and Medical Research Council (NHMRC) funded Reflexive Evidence and Systems interventions to Prevention Obesity and Non‐communicable Disease (RESPOND) (APP1151572). The opinions, analysis and conclusions in this paper are those of the authors and should not be attributed to the NHMRC. CVH is supported by a PhD scholarship from Deakin University Institute of Health Transformation.

## ETHICAL APPROVAL

Ethics approval was obtained from Deakin University Faculty of Health (HEAG‐H 192_2021). All participants provided written consent.

## Supporting information


Appendix S1.

Appendix S2.
