# Supplementary material for: Lived experience of participants who engaged in the co‐creation of initiatives to improve children's health in a rural Australian community
Source: Aust J Rural Health. 2023 May 18;31(4):659–69. doi: 10.1111/ajr.12996 (PMC10946477; doi:10.1111/ajr.12996)
Supplement: Supplementary file 1 — Appendix S1. [file AJR-31-659-s001.docx]

The Mansfield RESPOND project

Start of Block: Default Question Block

Q1 Select your age group

- 18-24 (1)
- 25-34 (2)
- 35-44 (3)
- 45-54 (4)
- 55-64 (5)
- 65-74 (6)
- 75 and over (7)

Q2 Select your gender

- Male (1)
- Female (2)
- Non-binary / third gender (3)
- Prefer not to say (4)

Q3 Select your role in the community (tick as many as relevant)

- Community stakeholder (1)
- Parent/carer of young children (2)
- School representative (3)
- Service club representative (4)
- Sports club representative (5)
- Health service representative (6)
- Local government representative (7)
- Other (8) ________________________________________________

Q5 Please tell us a little about your motivation (reasons) to join the Mansfield RESPOND project?

________________________________________________________________

Q6 Did you attend any of the Group Model Building (GMB) workshops? Which one(s)? (tick as many as relevant)

- None (1)
- GMB Workshop 1 (2)
- GMB Workshop 2 (3)
- GMB Workshop 3 (4)

Q7 In which stages of the Mansfield RESPOND project were you involved? (tick as many as relevant)

- Identification of the problem / Identification of relevant stakeholders for the project (1)
- Analysis of the causes of the problem (2)
- Definition of actions to address the problem (3)
- Design and planning the actions to address the problem (4)
- Implementing the actions to address the problem (5)
- Evaluating the actions to address the problem (6)

Q9 To what extent do you agree or disagree with the following statements about the Mansfield RESPOND Project?

|  | Strongly disagree (1) | Somewhat disagree (2) | Neither agree nor disagree (3) | Somewhat agree (4) | Strongly agree (5) |
| --- | --- | --- | --- | --- | --- |
| Useful for community stakeholders (1) |  |  |  |  |  |
| It helped to understand the interactions between participants (2) |  |  |  |  |  |
| It provided the means for stakeholders to share their experiences (3) |  |  |  |  |  |
| It allowed to build a platform to implement ideas and to continue the dialogue among stakeholders (4) |  |  |  |  |  |
| It allowed equal partnership between the people involved (5) |  |  |  |  |  |
| It allowed an open communication (6) |  |  |  |  |  |
| It maintained respect for all the participants (7) |  |  |  |  |  |
| It allowed an empathetic communication (8) |  |  |  |  |  |
| It allowed participants to design actions together (9) |  |  |  |  |  |
| It allowed participants to share their power to make changes (10) |  |  |  |  |  |
| It include all participant's perspectives and skills (11) |  |  |  |  |  |
| It respected and value the knowledge of every participant (12) |  |  |  |  |  |
| It allowed reciprocity (13) |  |  |  |  |  |
| It contribute to building and maintaining relationships between participants (14) |  |  |  |  |  |

End of Block: Default Question Block
