# Supplementary material for: Lived experience of participants who engaged in the co‐creation of initiatives to improve children's health in a rural Australian community
Source: Aust J Rural Health. 2023 May 18;31(4):659–69. doi: 10.1111/ajr.12996 (PMC10946477; doi:10.1111/ajr.12996)
Supplement: Supplementary file 2 — Appendix S2. [file AJR-31-659-s002.docx]

**Lived experience of co-design to improve children’s health in the rural Mansfield RESPOND project**

Focus Group Guide

Thank you for agreeing to participate in this focus group that will discuss your own experiences of working in the Mansfield RESPOND project.

My name is XX. I am a researcher from YY. [Background of researcher provided]

The information received from this focus group will not identify any participant but may use some quotes that you say during this discussion. We aim to use the information in a published manuscript, if this happens, you will each receive a copy of this publication if you want one.

We are on zoom, so I ask you all to ‘mute’ when not speaking, just to block out background noise, if you wish to speak next, please raise your hand (virtually or physically), if there is no one speaking, please just unmute and contribute your ideas to the discussion.

The focus of this discussion is on the ‘lived experience’ of the co-design or co-creation of local health initiatives – like the Mansfield RESPOND project. This means we want to hear from you about what has worked in the ‘planning and doing’ of Mansfield RESPOND from your own perspectives. I will provide some general questions to start the discussion, but we will not follow a structured list of questions. To clarify what we mean by ‘co-design’ or ‘co-creation’, we just mean that the community members of Mansfield, such as yourselves, decided what to do to help create a healthier Mansfield community, you weren’t asked to implement a program, you were asked about your ideas and encouraged to work on those with some support.

OK, let’s begin:

I’d like to start by opening the floor to some ideas of what has helped the Mansfield RESPOND project to get moving?

(Prompts if needed: were you involved from the beginning of RESPOND, how did you hear about RESPOND, do you value your involvement in RESPOND)

Gee, that’s some really great discussion there.

Some people have touched on a couple of things that perhaps could have been done better or could be done better in the future to make RESPOND stronger, does anyone have some ideas about that?

(Prompts if needed: how did volunteers get involved, were there barriers to getting ideas up and running?)

I really like the diverse insights that we are gathering from this discussion, thank you.

There are a couple more specific questions I’d like to ask as we get close to the end of this hour.

I wonder if anyone would like to share what ‘value’ or ‘values’ they may have gained from their participation in RESPOND? For example, often we ‘work’ to get paid, but most of you have ‘worked’ in RESPOND for other reasons, can you share some of these insights with us if you are comfortable to do so.

Finally, would you recommend other community members get involved with a co-design project and why or why not?

Thank you so much for your time. We really value your input.

Best wishes for a great day.
